# Effect of topical corticosteroids on nasal patency after acute positive airway pressure exposure

**DOI:** 10.1016/j.bjorl.2019.09.011

**Published:** 2019-11-03

**Authors:** Leonardo Balsalobre, Aline Bruno Figueiredo, Rogério Pezato, Reginaldo Raimundo Fujita

**Affiliations:** a Universidade Federal de São Paulo (UNIFESP), Departamento de Otorrinolaringologia/Cirurgia de Cabeça e Pescoço, São Paulo, SP, Brazil; b Universidade Federal de São Paulo (UNIFESP), São Paulo, SP, Brazil

**Keywords:** Nose, CPAP, Nasal obstruction, Allergic rhinitis, Topical corticosteroid

## Abstract

**Introduction:**

Nasal congestion and obstruction are reported in the majority of continuous positive airway pressure users and are frequently cited as reasons for noncompliance. Baseline inflammation due to allergic rhinitis could increase or exacerbate the inflammatory effect of high airflow in the nasal cavity as the result of continuous positive airway pressure and lead to greater continuous positive airway pressure intolerance. In this setting, intranasal steroids would be expected to counteract the nasal inflammation caused by allergic rhinitis and/or continuous positive airway pressure.

**Objective:**

The aim of the present study is to evaluate the effects of topical corticosteroid use on nasal patency after acute exposure to positive pressure.

**Methods:**

Ten individuals with allergic rhinitis were exposed to 1 h of continuous airway pressure (15 cm H_2_O) in the nasal cavity, delivered by a continuous positive airway pressure device. Visual analog scale, nasal obstruction symptom evaluation scale, acoustic rhinometry and peak nasal inspiratory flow were performed before and after the intervention. After 4 weeks topical nasal steroid (budesonide) application, positive pressure exposure was repeated as well as the first assessments.

**Results:**

Patients reported a statistically significant improvement both on the visual analog (*p* = 0.013) and obstruction symptom evaluation scales (*p* < 0.01). Furthermore, objective measurements were improved as well, with increased nasal cavity volume on acoustic rhinometry (*p* = 0.02) and increased peak nasal inspiratory flow (*p* = 0.012), after corticosteroid treatment.

**Conclusion:**

In patients with allergic rhinitis, intranasal corticosteroid therapy improved objective and subjective parameters of nasal patency after acute exposure of the nasal cavity to positive pressure.

## Introduction

Obstructive sleep apnea (OSA) is a highly prevalent disorder. In a large epidemiologic study conducted in a major Brazilian metropolis, it was found to affect 32.8% of the adult population.^1^ OSA is characterized by recurrent episodes of upper-airway obstruction occurring during sleep,^2^ combined with recurrent cycles of desaturation and reoxygenation, sympathetic overactivity and intrathoracic pressure changes, sleep fragmentation, decreased Quality of life (QoL), significant functional impairment, increased risk of road traffic accidents, and medical comorbidities (particularly cardiovascular). The first-line treatment of OSA is continuous positive airway pressure (CPAP), which has been shown to reduce the risks of the aforementioned complications, improve QoL, and lower the rate of motor vehicle accidents.^3^ Variable adherence to therapy, however, limits its overall effectiveness; 46–83% of patients are nonadherent.^4^ The user-mask interface, discomfort from the air pressure required, nasal obstruction and dryness, and psychological and social factors lead to poor acceptance and nonadherence.^5^

Allergic Rhinitis (AR) is a chronic mucosal inflammation with a prevalence of up to 25% in European Countries^6^ and could be one of the factors responsible for CPAP-related discomfort in patients with OSA. Although allergic rhinitis and OSA are closely associated, and each condition can be detrimental to the other,^7^ there is still no consensus on the actual effects of CPAP on the nasal cavity of patients with AR. A recently published article compared individuals with and without rhinitis and demonstrated a difference in physiological and biological changes in the nasal mucosa of those with OSA, but found no exacerbation of nasal symptoms.^8^ On the other hand, in a previous study, our group showed that individuals exposed to continuous positive pressure in the nasal cavity via CPAP experienced a deterioration of nasal symptoms, especially nasal obstruction. Furthermore, in individuals with allergic nasal symptoms, said deterioration is more severe than in patients without these symptoms.^9^ Thus, it is worth considering that baseline inflammation due to AR could increase or exacerbate the inflammatory effect of high airflow in the nasal cavity as the result of CPAP and lead to greater CPAP intolerance.

Treatment with intranasal corticosteroids is strongly recommended for patients with a clinical diagnosis of AR and symptoms that interfere with their daily life.^10^ As nasal complaints are a significant problem in patients with OSA who use CPAP, evaluating and treating such complaints is critical for the proper management of these patients.^8^ In this setting, intranasal steroids would be expected to counteract the nasal inflammation caused by AR and/or CPAP.

Within this context, the objective of the present study is to evaluate the effects of topical corticosteroid use on nasal patency after acute exposure to positive pressure.

## Methods

This study extended from January to March 2018 at the Department of Otolaryngology and Head and Neck Surgery, Federal University of São Paulo (UNIFESP), Brazil. The UNIFESP institutional Research Ethics Committee institutional Research Ethics Committee approved the project with protocol number 897.279 on 3 December 2015. All participants volunteered to take part in the study and provided written informed consent.

The sample included healthy individuals, aged 18–30 years, with no diagnosis or history of treatment for breathing-related sleep disorders. All were recruited through ads posted throughout the UNIFESP campus. After screening, each participant was interviewed to exclude those with factors that might influence nasal patency:

Use of medications such as antihistamines, antihypertensives, topical vasoconstrictors, systemic vasodilators, and topical and systemic corticosteroids in the preceding month; sinonasal infection and/or inflammation in the preceding month; history of tumors and/or prior sinonasal surgery; smoking or illicit drug use; and indigenous population.

Participants were also asked whether they experienced any nasal allergy symptoms, such as itching, sneezing, or nasal discharge. Those who replied positively underwent a skin prick test. Participants with a negative prick test were excluded from further analysis.

### Skin prick test

Prick tests were performed by inoculation of the right forearm in the morning and reading after 15 min. One needle was used for each antigen in each patient.

All allergen extracts had the same provenance (standardized) and the following allergies were tested:

Histamine

*Dermatophagoides pteronyssinus*

*Dermatophagoides farinae*

*Blomia tropicalis*

*Penicillium notatum*

*Alternaria alternata*

*Aspergillus fumigatus*

Dog

Cat

*Blattella germanica*

*Periplaneta americana*

Histamine served as a positive control. The test was considered positive when an allergen raises a wheal 3 mm or larger, the reaction is greater than or equal to that caused by histamine, and the patient has no reaction to the saline solution.

### Exposure to continuous positive airway pressure

All subjects were exposed to 1 h of continuous airway pressure (15 cm H_2_O) in the nasal cavity, delivered by a CPAP device (F&P Icon, Fisher & Paykel Healthcare Ltd., Auckland, New Zealand) attached to a nasal mask (Meridian Nasal Mask, ResMed Ltd., Bella Vista, Australia), while awake in the sitting position. No topical medications were placed in the nose before or after the intervention, and air leak through the mask was ruled out.

The following were performed and/or administered in all participants immediately before and after the use of CPAP:

Visual analogue scale (VAS) of nasal obstruction

Nasal Obstruction Symptom Evaluation (NOSE) modified scale;

Acoustic rhinometry (AcRh); and

Peak nasal inspiratory flow (PNIF) measurement.

### Visual analogue scale (VAS)

**Figure 1:**
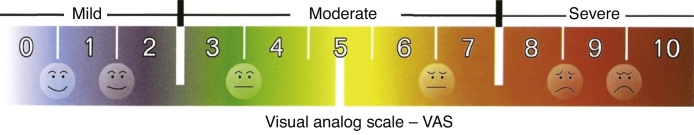
Visual analogue scale (VAS) of nasal obstruction.

To evaluate nasal obstruction, each participant was asked to score their nasal obstruction at that point in time on a scale ranging from 0 to 10, with 0 = absence of nasal obstruction and 10 = complete nasal obstruction (Fig. 1).

### Nasal obstruction symptom evaluation (NOSE) modified scale

We also administered an adapted version of the NOSE scale, in which the items referring to nasal obstruction during sleep and exercise had been removed, as individuals did not perform physical activity and did not sleep after CPAP exposure. For each of the three questions, scores could range from 0 to 4, for a maximum score of 12 points (Table 1).

**Table 1:** Nasal Obstruction Symptom Evaluation (NOSE) scale.

	Not a problem	Very mild problem	Moderate problem	Fairly bad problem	Severe problem
1. Nasal congestion or stuffiness	0	1	2	3	4
2. Nasal blockage or obstruction	0	1	2	3	4
3. Trouble breathing through my nose	0	1	2	3	4
4. Trouble sleeping	0	1	2	3	4
5. Unable to get enough air through my nose during exercise or exertion	0	1	2	3	4

### Acoustic rhinometry (AcRh)

For objective evaluation of nasal patency, participants underwent AcRh and PNIF measurement.

AcRh was performed without administration of vasoconstrictor. The test was conducted in an acoustically treated room with all factors necessary to ensure the accuracy of the procedure, as standardized by the international Standardisation Committee on Objective Assessment of the Nasal Airway. Each participant remained 30 min in an air-conditioned room (temperature set to 21 °C before measurement), and ambient humidity was kept in the 50–60% range, the head of each participant was stabilized to ensure proper positioning of the pulse tube, petroleum jelly was used to prevent air leak, and participants were instructed to control their breathing. To ensure the accuracy of the test, at least three curves were plotted for each nostril. After each measurement, the nosepiece was removed from the naris, reconnected, and a new measurement was then obtained. The results were deemed adequate if the coefficient of variability was lower than 10%. The recorded curves were used to obtain a mean curve for each naris. The values of these mean curves were then analyzed. The same investigator performed all examinations. The cross-sectional area between the distances 0 and 5 cm, expressed in cm^2^, was used for objective comparison of findings.

### Peak nasal inspiratory flow (PNIF)

Measurement of PNIF was performed with an In-Check Nasal Inspiratory Flow Meter (ClementClarke International, Harlow, UK), equipped with an air-cushioned facemask. The device consists of a small facemask connected to a plastic cylinder through which air flows during a forced inspiration. PNIF was measured three consecutive times with a 1 min interval between measurements, all acquired with the participant in the standing position. Results are obtained immediately, as with the peak expiratory flow meters used routinely in Pulmonology practice to assess the expiratory capacity of the lungs. The largest measure obtained was used for analysis.

### Use of intranasal corticosteroids

Once all baseline assessments had been completed, all patients received four bottles of budesonide 100 mcg and instructed to administer 1 spray into each nostril in the morning and evening. All participants were taught how to apply the spray properly.

After 28 days of budesonide administration, participants were re-exposed to CPAP. Before and after this exposure, the VAS and NOSE scales were re-administered and AcRh and PNIF measurement were performed again, exactly as before.

**Figure 2:**
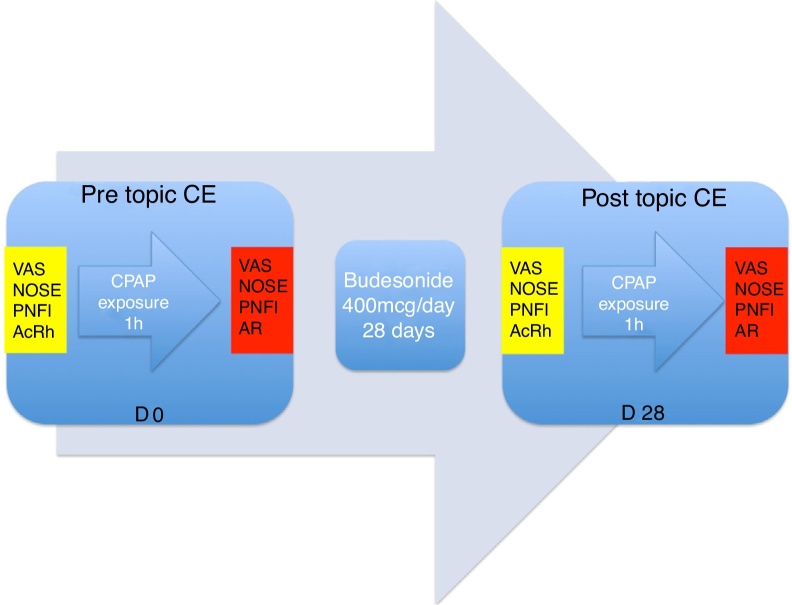
Study design. CE, corticosteroid; VAS, visual analogue scale; NOSE, Nasal Obstruction Symptom Evaluation scale; PNFI, peak nasal inspiratory flow; AcRh, acoustic rhinometry.

The study design is demonstrated in Fig. 2.

### Statistical analysis

Data were tabulated and analyzed in SPSS v. 22 (IBM Corp., New York, NY, USA) and Prism v. 7 (GraphPad Software Inc., La Jolla, CA, USA). The nonparametric Wilcoxon test was used to assess within-group differences before and after exposure to CPAP. In all cases, *p*-values <0.05 (95% CI), were deemed statistically significant.

## Results

The original sample comprised 20 subjects: 10 were excluded because they did not meet the inclusion and exclusion criteria. Thus, the final sample totaled 10 subjects. The mean age was 23.5 years, with 7 male and 3 female patients.

### Effect of topical corticosteroid before positive pressure exposure

VAS, NOSE, PNIF, and AcRh findings before and after the course of topical corticosteroids, all before CPAP exposure (yellow in Fig. 2), were compared.

On subjective evaluation of nasal symptoms (VAS and NOSE), participants reported significant improvement after 28 days of topical corticosteroid use (*p* = 0.02 and *p* = 0.016 respectively). Conversely, there was no improvement in objective parameters (PNIF and AcRh) after corticosteroid therapy, (*p* = 0.681 and *p* = 0.445 respectively) (Table 2).

**Table 2:** Descriptive statistics in 10 subjects (pre vs. post-corticosteroid use at pre-exposure to positive pressure)^a^.

	Pre-corticosteroid (D0)	Post-corticosteroid (D28)	*p*^b^
Visual Analogue Scale	3.7 ± 2.66 (0‒8)	1.8 ± 2.25 (0‒7)	0.02
Peak nasal inspiratory flow	113 ± 27.10 (70‒60)	121.5 ± 40.69 (50‒80)	0.681
Acoustic rhinometry	26.37 ± 9.16 (9.22‒41.1)	28.78 ± 14.96 (10.51‒60.59)	0.445
NOSE	4.5 ± 3.50 (0‒8)	0.9 ± 1.19 (0‒4)	0.016

NOSE, Nasal Obstruction Symptom Evaluation; SD, Standard Deviation.

a Values are mean ± SD (minimum–maximum).

b Wilcoxon test.

### Effect of topical corticosteroid after positive pressure exposure

Separate comparisons of VAS, NOSE, PNIF, and AcRh findings before and after topical corticosteroid use, but after CPAP exposure (red in Fig. 2), were performed.

**Figure 3:**
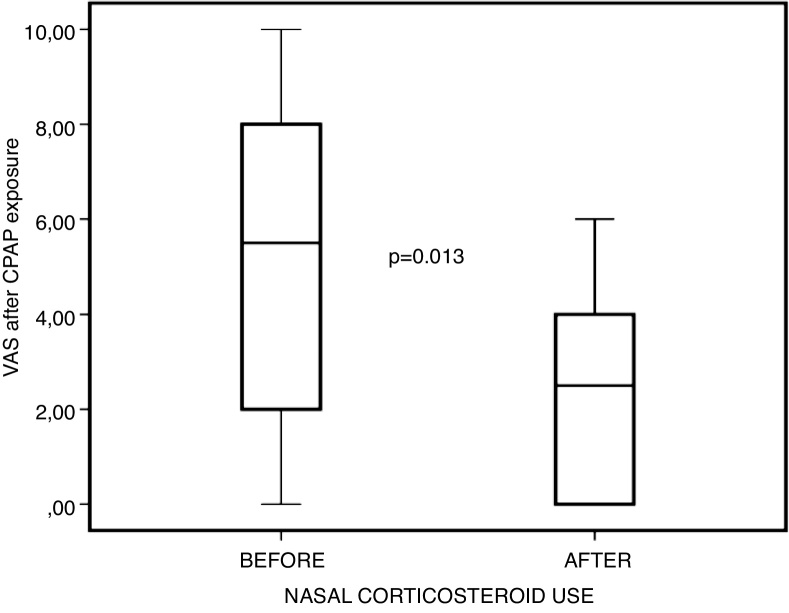
Comparison between visual analogue scale (VAS) scores before and after nasal steroid use, after positive pressure exposure.

**Figure 4:**
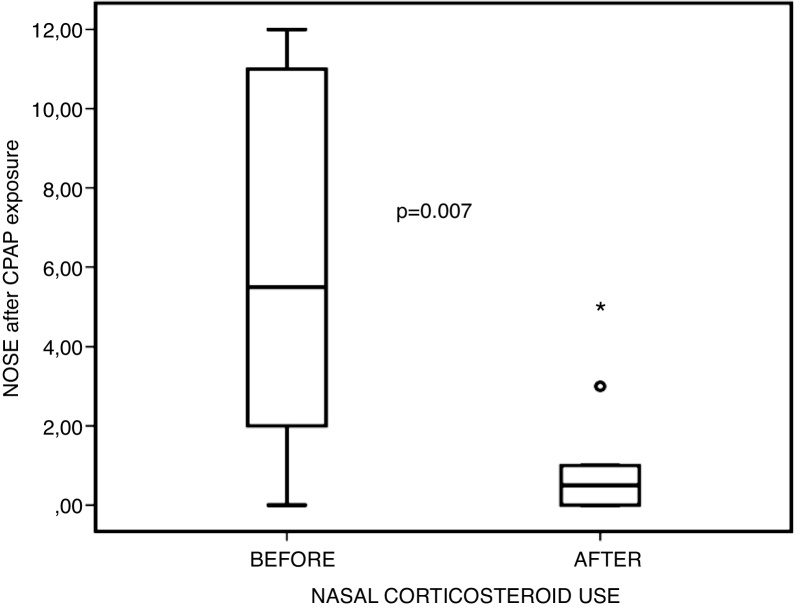
Comparison between NOSE scores before and after nasal steroid use, after positive pressure exposure.

**Figure 5:**
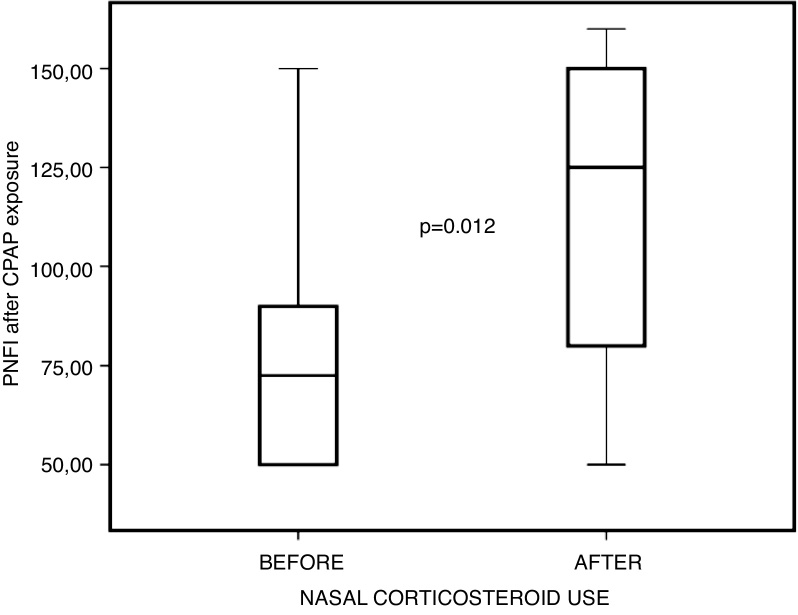
Comparison between PNIF values before and after nasal steroid use, after positive pressure exposure.

**Figure 6:**
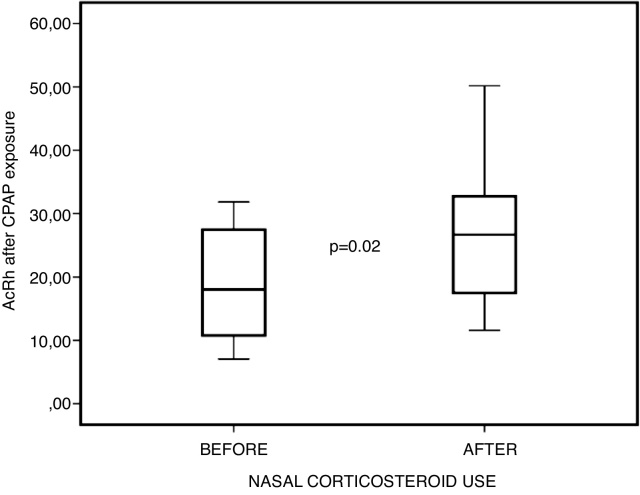
Comparison between AcRh findings before and after nasal steroid use, after positive pressure exposure.

After 4 weeks of topical corticosteroid therapy, patients reported improvement both on the VAS (Fig. 3) and NOSE (Fig. 4). Furthermore, objective measurements were improved as well, with increased nasal cavity volume on AcRh (Fig. 5) and increased PNIF (Fig. 6) (Table 3).

**Table 3:** Descriptive statistics in 10 subjects (pre vs. post-corticosteroid use at post-exposure to positive pressure)^a^.

	Pre-corticosteroid (D0)	Post-corticosteroid (D28)	*p*^b^
Visual Analogue Scale	5.1 ± 3.38 (0‒10)	2.4 ± 2.11 (0‒6)	0.013
Peak nasal inspiratory flow	82 ± 35.76 (50‒150)	114.5 ± 41.66 (50‒160)	0.012
Acoustic rhinometry	18.45 ± 8,82 (7.06‒31.81)	26.5 ± 12 (11.59‒50.17)	0.022
NOSE	6.3 ± 4.47 (0‒12)	1.1 ± 1.66 (0‒5)	0.007

NOSE, Nasal Obstruction Symptom Evaluation; SD, Standard Deviation.

a Values are mean ± SD (minimum–maximum).

b Wilcoxon test.

## Discussion

The present study demonstrated that the use of topical corticosteroids by individuals with AR led to an improvement in nasal patency (both objectively and subjectively measured) after acute exposure to positive pressure in the nasal cavity.

AR is a chronic inflammatory condition of the nasal mucosa. Studies have shown that exposure of the nasal mucosa of allergic and non-allergic patients to CPAP leads to an increase in nasal neutrophil levels.^8^, ^11^ A recent study by our group showed that exposure of the nasal cavity to CPAP led to deterioration of nasal parameters, especially nasal patency. This deterioration was even worse in individuals with nasal allergic symptoms.^9^ This leads us to believe that CPAP may exacerbate a baseline inflammatory state of the nasal mucosa in patients with AR, which in turn may potentiate the usual side effects of CPAP therapy. Some studies, however, maintain that CPAP itself produces a subclinical nasal inflammation because, despite a detected increase in neutrophils in the nasal cytology of CPAP users, there was no worsening of nasal symptoms in patients with AR.^8^ It is worth noting that these contradictory results may attributable to differences in methodology. Both the present study and the previous study by our group^9^ assessed nasal parameters immediately before and after CPAP exposure, while the aforementioned cytology study^8^ evaluated specific physiological and biological changes in the nasal mucosa of patients with OSA after chronic CPAP use rather than immediately after CPAP exposure. It can be hypothesized that the potential irritative effect of continuous pressure on the nasal mucosa, especially in individuals with AR, is more intense during CPAP therapy and, consequently, immediately after its use, and may become less symptomatic a few hours after exposure.

Topical corticosteroids are the first-line therapy of choice for AR. These drugs act locally on the nasal mucosa, primarily by regulating protein synthesis, leading to inhibition of various pro-inflammatory cytokine productions^12^ and, consequently, reduced nasal congestion. A recent randomized placebo-controlled study showed a significant improvement in nasal obstruction and AHI in patients with OSA following a 1 month course of intranasal fluticasone.^13^ Although our study did not include patients diagnosed with OSA, only subjects with AR, our initial evaluation of the effect of a 28 day course of intranasal budesonide showed a significant improvement in subjective nasal obstruction (VAS and NOSE), corroborating these findings. It is worth noting that there was no statistically significant improvement on post-treatment and pre-treatment comparison of these individuals through objective parameters (AcRh and PNIF).

However, when comparing nasal obstruction scores — both subjective (VAS and NOSE) and objective (AcRh and PNIF) — before and after the use of topical corticosteroids, but after exposure to acute positive pressure via CPAP, statistically significant improvement was observed in all these parameters. This leads us to believe that acute positive pressure exposure actually potentiates chronic irritation of the nasal mucosa, which in turn has been mitigated by topical corticosteroid therapy; this may even account for the improvement in objective measures of nasal patency, which was not observed in the previous comparison without exposure to CPAP.

Two randomized, placebo-controlled clinical trials^14^, ^15^ assessed the effects of topical nasal corticosteroid therapy (fluticasone) on CPAP compliance. Fluticasone was associated with some benefit on average duration of CPAP use per night, but statistical significance was not reached. Both studies recruited unselected groups of patients with OSA, with no specific focus on preexisting rhinitis or nasal symptoms. It is well known that the clinical response to nasal steroids is better in patients with AR than in those without AR.^16^ Accordingly, a systematic review and meta-analysis of this subject suggested that nasal steroids may improve adherence to CPAP use to a greater extent in patients with AR than in those with non-allergic rhinitis.^17^ The present study showed that topical nasal corticosteroids were effective in mitigating the acute effects of positive pressure on the nasal cavity of individuals with AR, especially nasal obstruction. It is important to note that the two studies cited above^14^, ^15^ evaluated the effect of topical nasal steroids on adherence to CPAP, while the present study consisted of objective and subjective evaluations of the effects of acute positive pressure exposure on the nasal cavity before and after use of topical budesonide.

One of the limitations of this study was the small number of patients. Nevertheless, it should be taken into account that allergic rhinitis affects 10–25% of the general population; therefore, we had to recruit volunteers who were willing to undergo an exhaustive battery of questionnaires and tests, had a positive skin prick test result, and were then subjected to two trials of CPAP. This constitutes another limitation: participants did not have sleep apnea, and their exposure to CPAP (for 1 h only, while awake, in the upright position) differed substantially from the typical exposure of an OSA patient (positive pressure for up to 8 h while sleeping and supine). Finally, the duration of intranasal steroid therapy (4 weeks) could be considered another potential weakness of this study. Nevertheless, we believe we obtained significant results.

## Conclusion

In patients with allergic rhinitis, intranasal corticosteroid therapy improved objective and subjective parameters of nasal patency after acute exposure of the nasal cavity to positive pressure.

## Conflicts of interest

The authors declare no conflicts of interest.
